# Deviation of the orientation angle of directional deep brain stimulation leads quantified by intraoperative stereotactic X-ray imaging

**DOI:** 10.1007/s10143-022-01801-8

**Published:** 2022-05-12

**Authors:** Josephiene M. Schmidt, Lars Buentjen, Joern Kaufmann, Doreen Gruber, Harald Treuer, Aiden Haghikia, Jürgen Voges

**Affiliations:** 1 Department of Neurology, University Hospital of Magdeburg, Leipziger Straße 44Saxony-Anhalt, 39108 Magdeburg, Germany; 2 Department of Stereotactic Neurosurgery, University Hospital of Magdeburg, Magdeburg, Germany; 3 Kliniken Beelitz, Movement Disorders Clinic, Beelitz-Heilstätten, Germany; 4 Department of Stereotactic and Functional Neurosurgery, Faculty of Medicine and University Hospital Cologne, University of Cologne, Cologne, Germany

**Keywords:** Deep brain stimulation, Directional stimulation, Lead orientation angle, Stereotactic X-ray

## Abstract

Directional deep brain stimulation (dDBS) provides multiple programming options. Knowledge of the spatial lead orientation is useful for time-efficient programming. Recent studies demonstrated deviations of up to 90° from the intended orientation angle. We examined the deviation of dDBS-lead orientation for leads from two different manufacturers using intraoperative stereotactic (STX) X-ray images. Intraoperative 2D-X-ray images were acquired after implantation of the first lead (TP1) and the second lead (TP2) enabling the estimation of the spatial position of the first lead at TP1 and TP2 and of changes of the orientation for a defined time period. Two investigators retrospectively estimated the orientation of the directional marker for 64 patients. The mean deviation from intended spatial orientation was 40.8° ± 46.1° for all examined leads. The spatial orientation of the first lead did not significantly change within a period of approximately 1 h. The degree of deviation did not differ significantly between two lead manufacturers but depended on the lead fixation technique. Our results showed deviations from the intended orientation angle immediately after the insertion of dDBS leads. The initial spatial orientation remained stable for approximately 1 h and was not caused by technical properties of the implanted lead. Hence, it was most probably the result of unintended mechanical torsion during insertion and/or fixation. Because precise determination of the lead orientation is mandatory for target-oriented dDBS programming, the use of additional imaging suitable for precise 3D visualization of lead contacts and/or the positioning marker is recommended.

## Introduction

Since the late 1980s, deep brain stimulation (DBS) has become an established treatment for selected patients with movement disorders such as idiopathic Parkinson’s disease (PD), dystonia, and essential tremor [1]. In PD, for instance, bilateral electrical stimulation of the subthalamic nucleus (STN) improves both motor symptoms and quality of life enabling a reduction of dopaminergic medication [2–4]. Up until recently, two different types of DBS leads are commercially available. The common lead contains 4 to 8 circular contacts in line. Monopolar electrical stimulation using one contact activates surrounding tissue covered by a spherical electrical field. The use of more than one contact per lead together with dedicated programming software enables the steering of the volume of tissue activated (VTA) along the longitudinal axis of the lead and/or the shape of this volume [5, 6].

By contrast, in directional leads, the two mid-level rings of the four rings are divided into three segments. Cathodic activation of all three segments enables stimulation with a spherical electrical field comparable to the common lead. Activation of only one or two segments creates an acentric field leading to directional current steering. Data from intra- and postoperative studies demonstrated that directional deep brain stimulation (dDBS) in comparison to conventional DBS (cDBS) widened the therapeutic window (higher threshold for side effects and a lower effect threshold) [7–9]. Additionally, bilateral STN-dDBS in PD patients was more effective than cDBS with respect to motor symptom improvement and reduction of dopaminergic medication [7].

In comparison to common DBS leads, individual testing of additional lead segments in a monopolar review increases the time necessary to work out optimal parameter settings [6, 10]. The information of the precise spatial orientation of the single segments could shorten the programming process as the VTA could a priori be adjusted according to the patient’s anatomy and clinical symptoms [11, 12]. Besides target point coordinates, and access angles, the rotation around the longitudinal axis of the lead (rotational angle) determines mainly the position of segments relative to the local anatomy at the target area. For determination of the rotational angle, directional leads are equipped with an X-ray marker close to the electrode tip. Several studies using imaging data from patients showed that there can be a significant difference between the intended and the final lead orientation [11, 13]. In addition, depending on the imaging modality and/or the access angle of the lead, determination of the rotational angle might be prone to errors [14]. Importantly, one in vivo study suggested that simple mechanical manipulation of the electrode during surgery might be responsible for electrode rotation [15].

With these findings in mind, we analyzed the electrode rotation of directional leads on intraoperative STX X-ray images in a large patient cohort. Primary question of this analysis was whether the initial orientation remains stable during a defined short, intraoperative observation period. Secondly, we aimed at to investigate whether leads from two different manufacturers may behave differently with respect to acute electrode rotation.

## Materials and methods

### Clinical data

We retrospectively analyzed the images of consecutive 64 patients (age at surgery 12–79 years; 34 male and 30 female patients) who underwent implantation of a dDBS-system at the Department of Stereotactic Neurosurgery of the University Hospital Magdeburg in the years 2017 to 2019. We implanted 84 Cartesia™ directional DBS leads (Boston Scientific, USA) in 42 patients and 44 directional leads of the St. Jude Medical Infinity™ DBS system (Abbott, USA) in 22 patients. Indications for deep brain stimulation included idiopathic Parkinson’s disease in 45 patients, tremor in 14 patients, and dystonia in 5 patients. Surgical targets for DBS were the subthalamic nucleus (STN) in 86 leads, the ventral intermediate nucleus (VIM) in 32 leads, and the globus pallidus internus (GPI) in 10 (overview in Table 1).

**Table 1:** Indications for dDBS lead implantation, target areas, and lead manufacturers

Diagnosis	Number of patients	STN	GPI	VIM	Boston Scientific	Abbott Infinity
Idiopathic Parkinson’s disease	45	43	0	2	33	12
Tremor	9	0	0	9	2	7
Dystonic tremor	3	0	0	3	0	3
Essential tremor (ET)	5	0	0	5	2	3
Overlap-syndrome ET and PD	1	0	0	1	0	1
Dystonia	5	0	5	0	5	0
Others	5	0	0	5	2	3
Multiple sclerosis	2	0	0	2	1	1
Multiple system atrophy	1	0	0	1	1	0
Unspecified postural and action tremor	1	0	0	1	0	1
Hereditary ataxia (SACS-mutation)	1	0	0	1	0	1
Total	64	43	5	16	42	22

### Implantation and imaging

All patients underwent preoperative MRI examinations on a 3 T-scanner according to standardized MRI-protocols comprising T1-, T2-, and PD-weighted series and DWI. DBS-surgery was performed by two neurosurgeons. For stereotactic implantation of brain electrodes, a modified Riechert-Mundinger stereotactic frame was mounted on the patient’s head and an intraoperative stereotactic CT examination was performed. We used dedicated software for image processing, co-registration of preoperative MRI-images, and treatment planning (prior to 2019: Praezis Plus 3.1, since 2019 iPS 6.0, inomed, Emmendingen, Germany).

Brain electrodes (Cartesia™ directional DBS lead DB 2202, Boston Scientific, USA; directional lead 8 channel St. Jude Infinity™ DBS system, Abbott, USA) were implanted under fluoroscopic guidance. Fluoroscopy monitored the introduction of the electrode into the brain, in particular the penetration depth and a possible deviation from the prescribed trajectory as depicted on the plane. Implantation was performed with the intention of adjusting the orientation radiopaque marker of the lead anteriorly. The electrodes were fixed at the skull surface using titanium miniplates (TM-fixation) or a titanium clip which was fixed into the burr hole by methylacrylate (TC-fixation). The electrode position was documented on intraoperative stereotactic X-ray images in a lateral and a frontal view. These images taken after implantation of the first lead and the second lead, respectively, provided stereotactic coordinates for both the target and the entry point of the lead. X-ray tubes permanently installed in the operating room ensured that the central beam did always hit orthogonally the center of the stereotactic frame and hence the zero plane of the 3D stereotactic coordinate system. All patients underwent postoperative non-stereotactic CT-examinations. These images were finally co-registered with the stereotactic CT- and X-ray images.

### Assessment of lead orientation using STX-XR

Two blinded raters (L.B., J.S.) estimated the electrode orientation on intraoperative, stereotactic X-ray images using the X-ray marker on the distal part of the electrode. The X-ray images were not chronically ordered and presented anonymously. We defined the orientation of the marker towards rostral (frontal) direction as 0° and towards posterior (occipital) direction as 180°. Medial rotations were labeled as “-values,” lateral rotations accordingly as “ + -values,” with increments of 5°. As a visual aid, we used a true to scale 3D-model of both Boston Scientific and Abbott Infinity dDBS leads with a marker and segmented contacts. The 3D-model could be manually rotated by the investigators facilitating the understanding of the perspective and hence guiding the estimation of the lead orientation angle on X-ray images.

For the analysis, the raters used X-ray images from two different time points. Images from time point 1 (TP1) documented the position of the first implanted lead and images from time point 2 (TP2) the position of both the first and the second implanted lead. The raters determined the orientation of the first implanted electrode for TP1 and TP2 and the orientation of the second implanted lead at TP2. In addition, we analyzed the lead orientation of 64 Boston Scientific leads using dedicated software developed by Sitz et al., which was validated for Boston Scientific directional leads only [12]. Using X-ray images registered to the stereotactic space, this software quantifies the actual lead orientation angle by comparing the measured images with a set of virtual marker images at different orientation angles (range 0° to 355°).

To improve the validity of the intended calculation, we determined intrarater reliability with two runs per rater at least 1 week apart. For the second run, only a random subsample was assessed instead of the total sample [16].

In summary, we had collected the following data:

1. Estimated lead orientation of the first lead directly after its insertion at TP1
2. Estimated lead orientation of the first lead after insertion of the second lead at TP2
3. Estimated lead orientation of the second lead directly after its insertion at TP2
4. Measured lead orientation for 64 dDBS leads (manufacturer: Boston Scientific) at TP2

### Statistics

For statistical analysis, we used SPSS Statistics 27 and Microsoft Excel. The Institute for Biometry and Medical Informatics of the University Hospital Magdeburg supervised the methodological procedure and controlled the results.

We calculated inter- and intrarater reliabilities as a measure for homogeneity and consensus between the two investigators, the investigators themselves, and between the investigators and the measured orientation angles. A two-way mixed model with absolute agreement of the intraclass-correlation coefficient (ICC) was used to calculate inter- and intrarater reliability and 95% confidence intervals. We determined the agreement of estimated and measured orientation angles in the same way. An intraclass correlation coefficient between 0.75 and 0.9 was defined as good agreement. We defined results above 0.9 as excellent agreement and values between 0.5 and 0.75 as moderate agreement. Results under 0.5 were defined as poor agreement [17, 18].

Since the interrater reliability was moderate, we averaged the values as estimated by the two investigators for each brain electrode and used the resulting mean values for further statistical analysis. Finally, mean, median, minimum, maximum, 25% and 75% quartile, and standard deviations of lead orientation directly after insertion were calculated for the first and second lead. In addition, we stratified the values with respect to different manufacturers (Abbott vs. Boston) and the fixation technique (TM-fixation vs. TC-fixation). Box-and-Whisker plots were used for graphical depiction of the data. The Wilcoxon signed-rank test was selected to examine whether the progression of deviation between TP1 and TP2 was significant or not. To determine the impact of different manufacturers of the fixation technique on the deviation of lead orientation, we applied a Mann–Whitney *U* test. For both statistical tests, a *p*-value of < 0.05 was deemed significant.

## Results

### Intrarater reliability

Calculation of the intrarater reliability is based upon a sample size in the second run of 21 patients for investigator 1 and assessment of the complete patient cohort in the second run for investigator 2. For investigator 1, the ICC for the first lead was 0.743 (95% confidence interval 0.463–0.888) and for the second lead 0.993 (95% confidence interval 0.983–0.997) indicating a moderate intrarater reliability for the first and an excellent intrarater reliability for the second implanted lead. Calculation of the ICC after withdrawal of an outlier improved the ICC of the first lead to 0.926 (95% confidence interval 0.823–0.970) improving the intrarater reliability from “moderate” to “excellent.” For investigator 2, the ICC for the first lead was 0.866 (95% confidence interval 0.798–0.917) and for the second lead 0.987 (95% confidence interval 0.978–0.992) indicating an excellent intrarater reliability for investigator 2.

### Interrater reliability

At first, we determined the interrater reliability of the two investigators by calculating the intraclass correlation coefficient (ICC) separately for the first and second lead. The ICC was 0.783 (95% confidence interval 0.651–0.878) for the first lead and 0.721 (95% confidence interval 0.564–0.840) for the second lead indicating moderate agreement between investigators.

Intraclass-correlation was also used to calculate the agreement of the determination of electrode orientation between investigators and computer-based analysis [12] including data from 64 dDBS leads of the manufacturer Boston Scientific. For the first lead, the ICC of investigator-based and computerized analysis was 0.676 (95% confidence interval 0.436–0.827) and for the second lead 0.606 (95% confidence interval 0.337–0.785). Both values indicated a moderate agreement between the two evaluation methods.

### Deviation of spatial electrode orientation

**Fig. 1:**
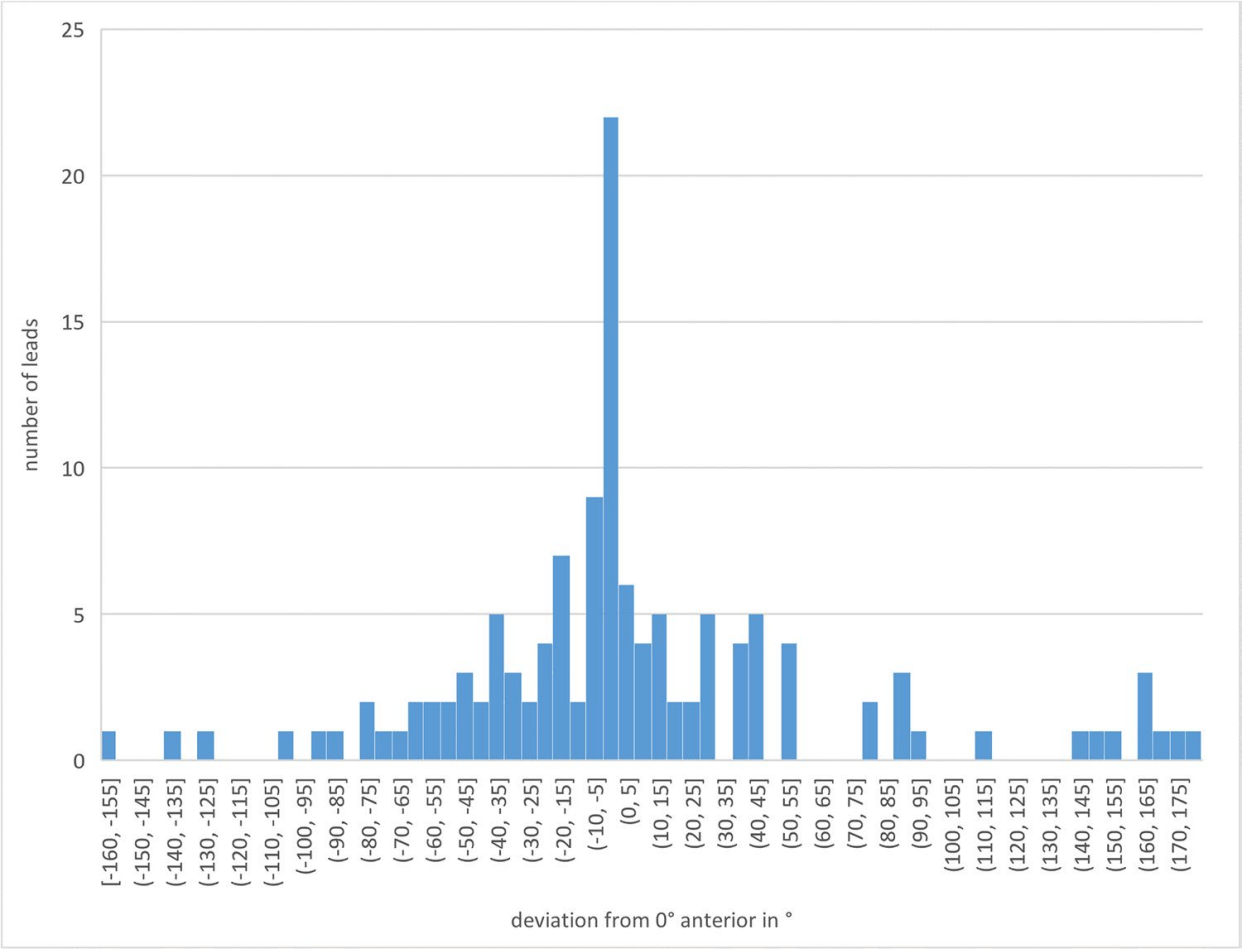
Histogram of the orientation angle with respect to the intended orientation (0° anterior) and direction of lead rotation

**Fig. 2:**
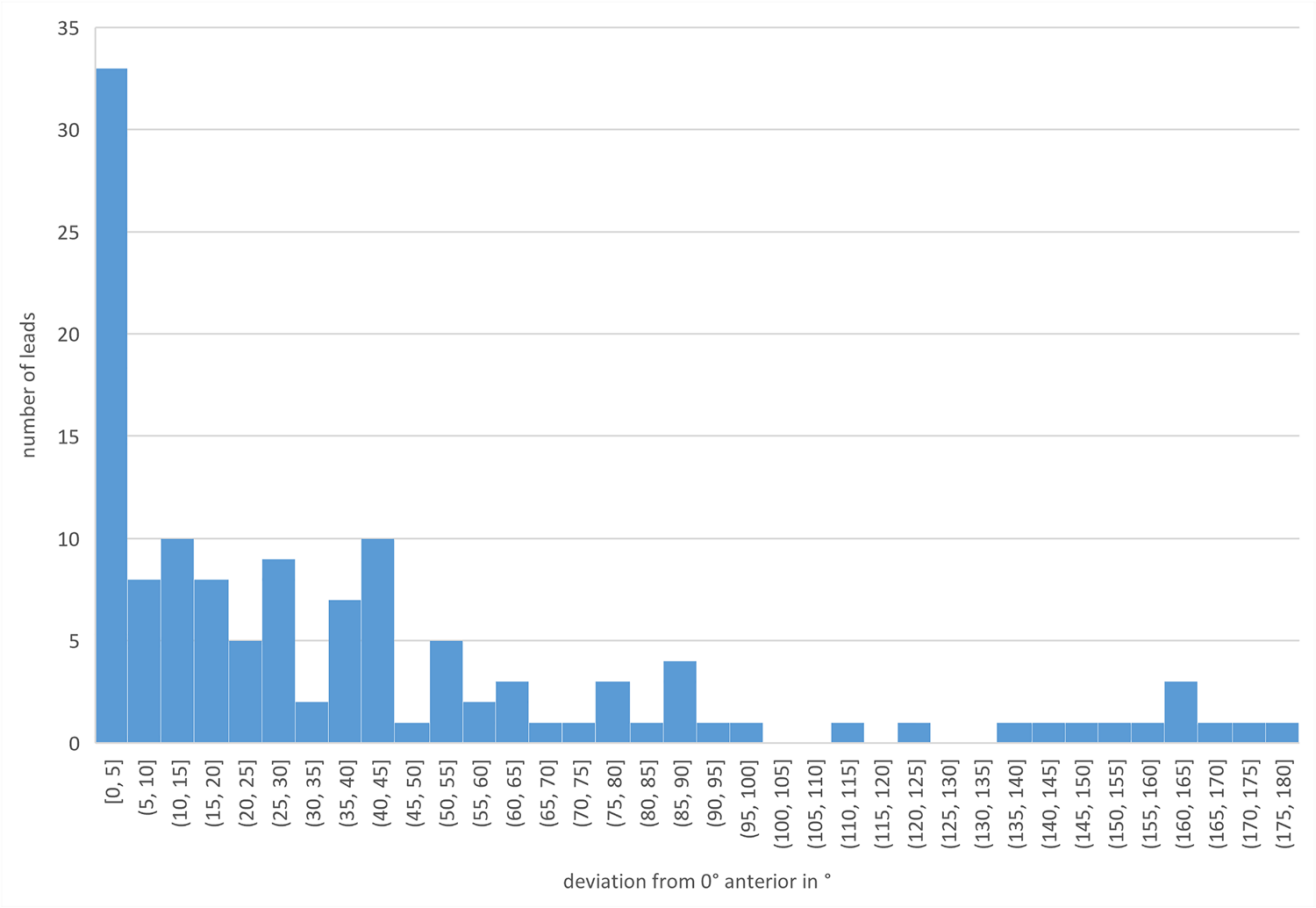
Histogram of the orientation angle with respect to the intended orientation (0° anterior) irrespective of the direction of lead rotation

**Fig. 3:**
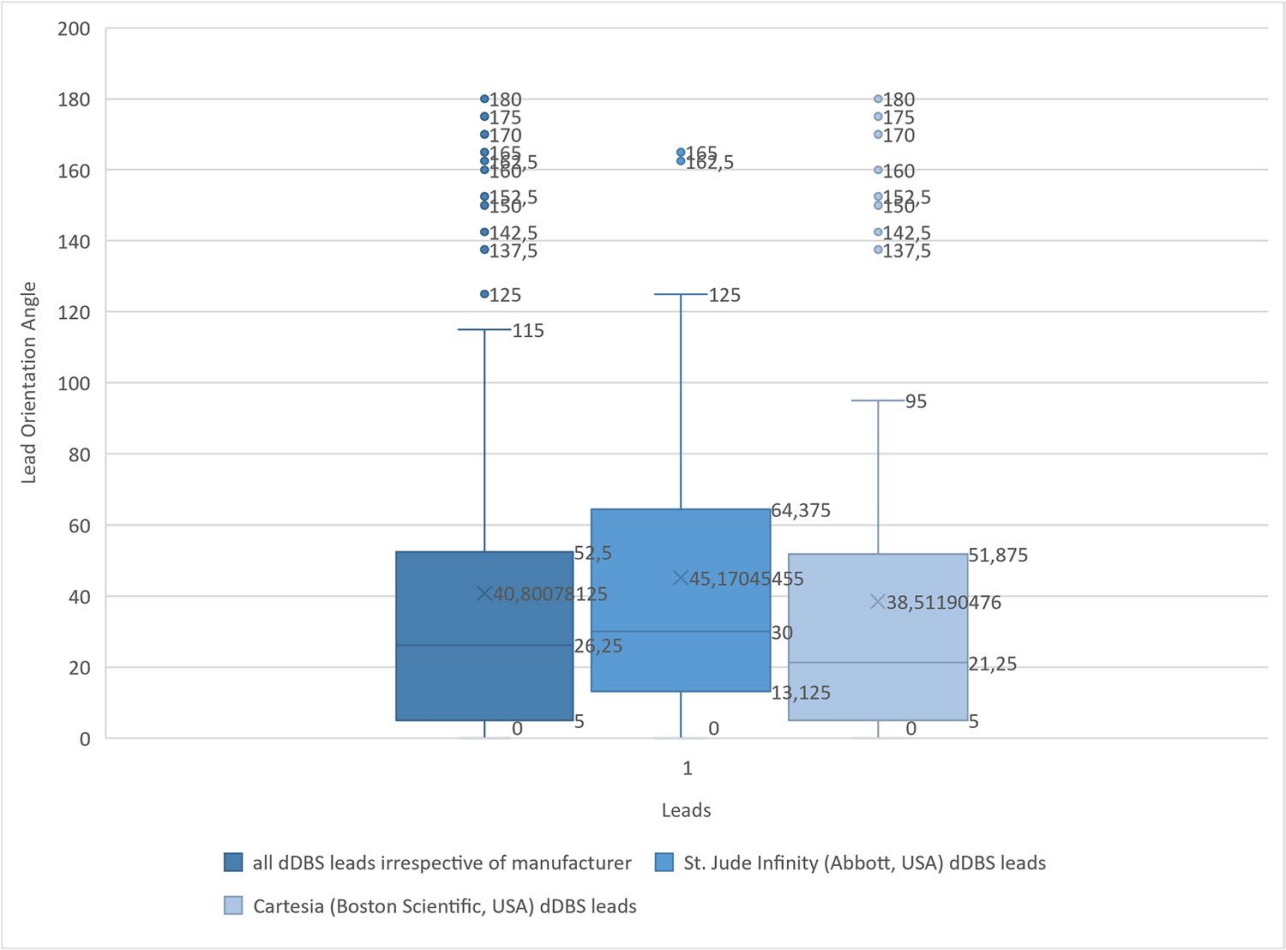
Boxplots depicting the orientation of all implanted leads. In addition, the orientation is stratified according to the two different lead models (St. Jude-Infinity or Boston-Cartesia dDBS leads)

The mean deviation between intended and final spatial orientation was 40.8° ± 46.1° for all examined leads (*N* = 128) with a maximum deviation of up to 180° (shown in Figs. 1, 2, 3). The mean deviation between intended and final spatial orientation of all leads manufactured by Abbott Infinity (*N* = 44) was 45.2° ± 46.1° with a maximum deviation of 165° (shown in Fig. 3). For all leads manufactured by Boston Scientific (*N* = 84), the mean deviation of intended and final spatial orientation was 38.5° ± 46.2° with a maximum deviation of 180° (shown in Fig. 3). For 64 Cartesia dDBS leads, computerized analysis showed a mean deviation between intended and final spatial orientation of 55.6° ± 51.2° with a maximum deviation of 175°.

The difference of the spatial orientation of the first lead between TP1 and TP2 (average observation time 60 min) was statistically not significant (*N* = 64, *Z* =  − 0.11, *p* = 0.916, Wilcoxon-signed-rank-test). In addition, the lead manufacturer had no significant impact on the degree of deviation (group A (*N* = 44), group B (*N* = 84); Mann–Whitney *U* test, *U* = 1629.5, *Z* =  − 1.098, *p* = 0.272).

A Mann–Whitney *U* test was calculated to determine a possible impact of lead fixation on the degree of rotation. Leads secured with the TC-technique (*N* = 64) rotated in the mean by 28.3° ± 29.7°. In contrast, those fixed with titanium plates (TM-technique, *N* = 64 leads) had a mean lead orientation of 53.3° ± 55.5°. This difference was statistically significant (*U* = 1558.0, *Z* =  − 2.339, *p* = 0.019).

## Discussion/conclusion

The current study is to our knowledge the first analysis of the orientation of dDBS leads for a defined, short time period in a larger cohort of patients using stereotactic intraoperative X-ray images. The results demonstrated that compared to the intended orientation angle (0° anterior), dDBS leads rotated immediately after the implantation in average by 40.8° around their longitudinal axis. This deviation appeared to be independent of the lead manufacturer. After an average observation time of 60 min, additional electrode rotation occurred but was not significant.

The dimension of rotation observed in the actual study was within the range as reported by other study groups. Using rotational fluoroscopy several weeks to months after implantation, deviations from the intended orientation up to 30° were registered [6]. Krüger et al. reported a median deviation of 26.5° (5.5–62.0°) for a time period of 108–189 min (intraoperative position compared to postoperative CT), when only the marker orientation was used [13]. In the study of Dembeck et al., postoperative CT scans (CT examination approx. 24–72 h postoperatively) displayed deviations of up to 90° from the intended electrode rotation with deviations of more than 30° in 42% of the leads and deviations of more than 60° in about 11% of the leads [11]. In the latter study, the factors “neuroanatomical target” and “stereotactic frame” had no impact on the degree of deviations. However, the deviation increased depending on the type of microdrive used for intraoperative microelectrode recording and dDBS lead implantation. This observation could be taken as a hint that mechanical manipulation of dDBS leads such as torsion might substantially contribute to rotational deviations [11].

Rau et al. simulated in a cadaver study an electrode implantation with torsion. After implantation in a sheep brain, the standardized 180° or 360° clockwise rotation of dDBS leads at the level of the skull (lead entry point) was not transferred to the lead tip by exactly the same angle of rotation. Instead, 3D rotational fluoroscopy images taken immediately after electrode manipulation displayed lower mean rotational deviations as anticipated of only 83.5° and 201°, respectively. After 24 h, mean rotational differences were 114.0° for the intended 180° clockwise rotation and 215.7° for the intended 360° clockwise rotation. These results showed as hypothesized by the investigators that independent from biological factors such as brain shift, mechanical manipulation could have a substantial effect on electrode rotation. In addition, neither time course nor the degree of rotation seemed to be predictable [15].

The time course of lead rotation has been discussed controversially. In the actual analysis, additional rotation registered at TP2 (approx. 60 min after implantation of the first lead) was not significant if compared to TP1 indicating that unintended lead rotation is a very early event. This assumption is in line with the observation of other studies, which reported stable positioning for time latencies of 4–9 days or a median time latency of 82 (range 1–811) days [11, 13, 19].

One important point with conceivable effects on electrode rotation could be the lead fixation technique used [13]. Unintended lead rotation due to mechanical manipulation of the electrode was most likely the reason for the deviation observed in the here analyzed patient cohort, because subgroup analysis demonstrated statistically significant different degrees of lead rotation depending on the routinely used fixation techniques.

Biological factors such as edema, CSF-loss, and/or pneumocephalus may also contribute to lead rotation. In the past, these factors and their possible impact on the rotational angle might not have been systematically evaluated or were not explicitly mentioned in published clinical studies. On the immediate postoperative CT-scans of the patients considered for the current analysis, no patient showed space-occupying hemorrhage or pneumocephalus.

The contour of the stereotactic marker along the lead axis differs to some extend from manufacturer to manufacturer, which could theoretically affect the accuracy of lead placement and/or the estimation of electrode rotation. However, in the current study, the difference between patients implanted with Cartesia leads and those treated with Infinity leads was not statistically significant, ruling out a major design effect.

A limiting factor of the current study is that the electrode rotation was determined using the marker orientation only, which is less precise compared to other methods. Krüger et al. determined the anterior orientation position of 32 dDBS leads on intraoperative X-rays (lateral view) using primarily the marker. In cases with adequate visualization of the marker, they applied additionally the “iron sight” (Isi) method introduced by Reinacher et al. [13, 20]. Compared to postoperative CT-scans (latency 108–189 min), the median electrode rotation was 1.5° (range 0.5–6.0°) when the Isi-method could be applied intraoperatively (9 leads). The rotation increased to 15.5° (9.5–35.0°) when the Isi-sign was not visible on intraoperative images and to 26.5° (range 5.5–62.0°) when only the marker could be identified [13].

## Conclusion

Our results showed deviations from the intended orientation angle due to electrode rotation immediately after the insertion of dDBS leads regardless of the manufacturer of the lead. The initial spatial orientation remained stable for approximately 1 h. Taking into consideration that the degree of rotation depended on the applied fixation technique, this observation suggests that lead rotation was most probably caused by unintended mechanical torsion during insertion and/or fixation and not by technical properties of the implant or biological factors. As a consequence, using only the positioning marker together with fluoroscopy as intraoperative guidance, it seemed not possible to control the rotational lead position mechanically with sufficient accuracy. Precise determination of the lead orientation, however, is mandatory for target-oriented current output, because it will shorten the time necessary for the postoperative adjustment of stimulation parameters. Hence, additional imaging particular suitable for precise 3D visualization of lead contacts and/or the positioning marker such as a CT-imaging-based sequential algorithm (DiODe), or the the “iron sight” (Isi) method [13, 14, 20, 21] is recommended.

## Data Availability

All data generated or analyzed during this study are included in this article. Further enquiries can be directed to the corresponding author.
